# iAMP-Attenpred: a novel antimicrobial peptide predictor based on BERT feature extraction method and CNN-BiLSTM-Attention combination model

**DOI:** 10.1093/bib/bbad443

**Published:** 2023-12-05

**Authors:** Wenxuan Xing, Jie Zhang, Chen Li, Yujia Huo, Gaifang Dong

**Affiliations:** School of Computer Science and Engineering, Northeastern University, No.195 Chuangxin Road, Hunnan District, Shenyang 110170, China; College of Computer and Information Engineering, Inner Mongolia Agricultural University, No.29 Erdos East Street, Saihan District, Hohhot 010011, China; School of Computer Science and Engineering, Northeastern University, No.195 Chuangxin Road, Hunnan District, Shenyang 110170, China; College of Computer and Information Engineering, Inner Mongolia Agricultural University, No.29 Erdos East Street, Saihan District, Hohhot 010011, China; College of Computer and Information Engineering, Inner Mongolia Agricultural University, No.29 Erdos East Street, Saihan District, Hohhot 010011, China

**Keywords:** antimicrobial peptides, BERT, CNN-BiLSTM-Attention, AMP predictor

## Abstract

As a kind of small molecule protein that can fight against various microorganisms in nature, antimicrobial peptides (AMPs) play an indispensable role in maintaining the health of organisms and fortifying defenses against diseases. Nevertheless, experimental approaches for AMP identification still demand substantial allocation of human resources and material inputs. Alternatively, computing approaches can assist researchers effectively and promptly predict AMPs. In this study, we present a novel AMP predictor called iAMP-Attenpred. As far as we know, this is the first work that not only employs the popular BERT model in the field of natural language processing (NLP) for AMPs feature encoding, but also utilizes the idea of combining multiple models to discover AMPs. Firstly, we treat each amino acid from preprocessed AMPs and non-AMP sequences as a word, and then input it into BERT pre-training model for feature extraction. Moreover, the features obtained from BERT method are fed to a composite model composed of one-dimensional CNN, BiLSTM and attention mechanism for better discriminating features. Finally, a flatten layer and various fully connected layers are utilized for the final classification of AMPs. Experimental results reveal that, compared with the existing predictors, our iAMP-Attenpred predictor achieves better performance indicators, such as accuracy, precision and so on. This further demonstrates that using the BERT approach to capture effective feature information of peptide sequences and combining multiple deep learning models are effective and meaningful for predicting AMPs.

## INTRODUCTION

With the gradual failure of traditional antibiotics in the face of the challenge of drug resistance, antimicrobial peptides (AMPs) have become a novel candidate for anti-infective drugs due to their ability to help organisms resist infections from bacteria, fungi, viruses and other microorganisms [1–3]. AMPs are a type of small molecule protein fragments that naturally exist in organisms [4, 5]. In addition to their anti-microbial properties [6], some studies [7, 8] have indicated that certain AMPs may have anti-proliferative, inducing apoptosis and inhibiting angiogenesis effects on cancer cells. Therefore, AMPs have provoked substantial attention in the fields of medicine and biomedical research [9, 10]. The accompanying problem of AMPs recognition and investigation [11, 12] also has become a wide-ranging preoccupation for researchers both domestically and internationally.

Due to the substantial human, material, financial and temporal resources required by traditional experimental methods for AMPs identification, many computational techniques have emerged in a plethora of works related to constructing AMPs databases and discovering AMPs [13, 14]. Research efforts in the establishment of AMPs databases such as APD3 [15], DBAASP [16], CAMP [17], YADAMP [18], SATPdb [19], dbAMP [20], among others, have been introduced with the aim of enabling researchers and scientists to gain deeper insights into the characteristics of these molecules and to provide data support for prediction purpose. Additionally, one recent development in this field is the Peptide Utility (PU) search server [21], which significantly streamlines peptide sequence searches across multiple AMPs databases, enhancing research efficiency in the study of AMPs. In the realm of AMP discovery research, peptide feature selection strategies, machine learning classifiers and deep learning predictors can facilitate researchers in efficiently screening and predicting potential AMP candidates. Among these, peptide feature selection strategies extract meaningful information from amino acid sequences to aid in distinguishing between AMPs and non-AMPs. For instance, AVPpred predictor [22] not only employs amino acid composition and physicochemical properties to extract valuable features from both anticancer peptides (ACPs) and non-ACPs but also subsequently utilizes machine learning algorithm for efficient prediction of ACPs. In addition to the aforementioned peptide selection strategies, methods such as pseudo amino acid composition (PseAAC) and composition, transition and distribution (CTD) are frequently adopted to depict the feature representation of protein or AMPs sequences [11]. As for machine learning classifiers, such as Support Vector Machine (SVM), Random Forest (RF), K-Nearest Neighbor (KNN) and others, leverage features extracted from peptide sequences for intelligent prediction and discern the antimicrobial potential of peptides. For example, [1] introduces a classifier called AMPfun based on the RF algorithm to differentiate between AMPs and their diverse functional activities. Similarly, iAMP-2L predictor [23] is developed utilizing the PseAAC and fuzzy KNN technique with the aim of facilitating the design of novel and highly effective antimicrobial agents. [24] and [25] employ the SVM algorithm to enhance the predictive performance of ACPs. [26] proposes the ACP_MS predictor, which is constructed using the monoMonoKGap method and AdaBoost model, to enhance the accuracy of identifying ACP sequences. Moreover, a multitude of deep learning models have emerged to drive the discovery and investigation of AMPs in recent years. For instance, sAMPpred-GAT [27] is the first work that utilizes the predicted peptide structures and graph attention networks (GAT) to improve the recognition performance of AMPs. [28] combines convolutional neural networks (CNN) and long short-term memory (LSTM) models to build a classifier aimed at addressing the AMPs identification challenge. ACP-GCN framework [29] based on graph convolutional networks (GCN) is proposed to effectively differentiate ACPs from non-ACPs sequences. In addition to the approaches mentioned above, bidirectional LSTM (BiLSTM) and recurrent neural networks (RNN) are also extensively employed to expedite the classification of novel AMPs as demonstrated by these researches [30–35].

In the past few years, transformer architecture [36] has achieved exceptional performance in a wide range of natural language processing (NLP) tasks owing to its ability to overcome the limitations encountered by traditional RNN and CNN when dealing with long-distance dependencies. Furthermore, transformer model is effectively applied to the tasks aimed at extracting feature information from peptide sequences and enhancing the accuracy of AMPs identification [37]. In comparison with conventional unidirectional transformer pre-trained model, bidirectional encoder representation from transformers (BERT) architecture [38] demonstrates better performance in a lot of tasks due to its capability for bidirectional context modeling. It is understood that the BERT method has been successfully utilized in the fields of protein lysine crotonylation (Kcr) [39], AMP classification [40–42], etc.

Previous researches have demonstrated the remarkable performance of BERT in a wide array of NLP tasks as well as in the domain of AMPs recognition. Inspired by these works, we introduce a new iAMP-Attenpred predictor built upon the BERT model and several deep neural network frameworks. It is worth noting that iAMP-Attenpred identification model not only extracts useful and important feature information of peptide sequences through BERT technology, but also is the first attempt to synergistically integrate CNN, BiLSTM and attention mechanism for the purpose of predicting AMPs sequences. In order to validate its effectiveness, we evaluate the capability of iAMP-Attenpred model to discern whether a given peptide sequence exhibits antimicrobial property. Experimental results indicate that our new design achieves significantly more dependable enhancements in AMPs sequences recognition when compared to the state-of-the-art predictors.

## MATERIALS AND METHODS

**Figure 1:**
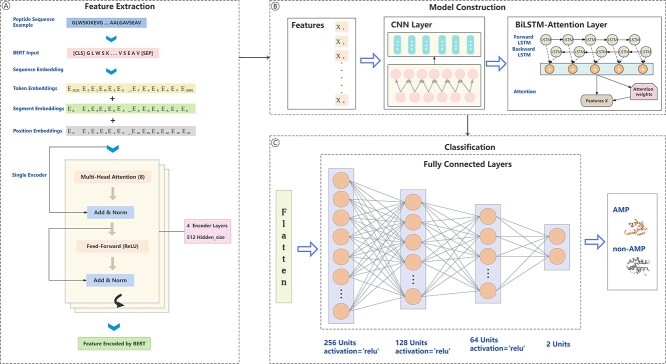
Overall architecture of iAMP-Attenpred predictor.

In order to help construct a serviceable prediction model for AMPs, we draw a flow chart for this work as shown in Figure 1. The specific details of each process in this flowchart are described in the following sections except for the Benchmark datasets section.

### Benchmark datasets

**Figure 2:**
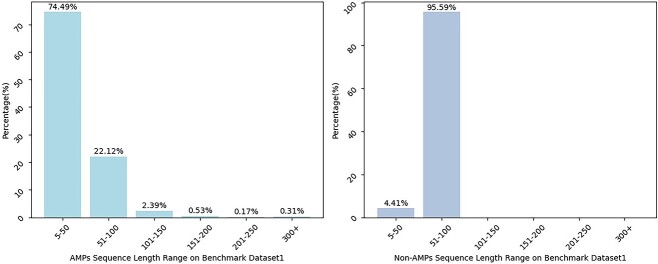
Percentage distribution histogram of sequence length ranges based on benchmark dataset1.

**Figure 3:**
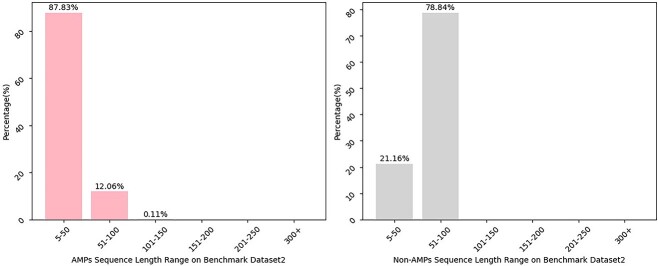
Percentage distribution histogram of sequence length ranges based on benchmark dataset2.

In this study, we select two datasets as benchmark datasets so as to achieve a fairer and more comprehensive comparison of performance with the existing approaches. These two datasets are obtained from these works [23, 43], and we label them as benchmark dataset1 and benchmark dataset2, respectively. The benchmark dataset1 consists of AMPs and non-AMPs sequences, where the AMPs data are collected from AMPer [13], APD3 [15] and ADAM [44] databases. To ensure the biological rationality and interpretability of AMPs dataset, sequences containing any codes beyond the 20 natural amino acid encodings are excluded. Meanwhile, one sequence from any pair of AMPs with the sequence similarity exceeding 90% is removed using the CD-HIT program [45] for the purpose of reducing redundant information. Additionally, the non-AMPs sequences are collected from the UniProt database [46] with a constraint that the residues length of protein fragments ranged from 5 to 100. And then the sequences containing any of these annotations, such as ’Antimicrobial’, ’Antibiotic’, ’Fungicide’ or ’Defensin,’ are excluded. The sequences that include codes beyond the 20 natural amino acids are also continuously eliminated. Moreover, the threshold of the CD-HIT program is set to 40% so as to mitigate the impact of homology on the analysis. As for the benchmark dataset2, the acquisition process of non-AMPs dataset is similar to the benchmark dataset1. Its AMPs sequences are obtained from APD database [47] and the CD-HIT program with the 40% threshold is utilized. The specific information of the two benchmark datasets finally obtained is shown in Table 1. At the same time, statistical approach is used for understanding the sequence length distribution of two benchmark datasets in our work. As shown in Figures 2 and 3, most AMPs and non-AMPs have sequence lengths ranging from 5 to 100 whether it is benchmark dataset1 or benchmark dataset2.

**Table 1:** Summary statistics information of two benchmark datasets

Datasets	Positive samples	Negative samples
Benchmark dataset1	3594	3925
Benchmark dataset2	879	2405

The choice of these two datasets in our study is based on several factors. Firstly, these datasets are widely recognized in the field and have been used in prior researches, which allows for meaningful comparisons with existing works. Additionally, these datasets are of high quality and reliability. Finally, these datasets are publicly available, which means that other researchers can replicate our work and validate our results. Therefore, their availability and suitability for our study make them the preferred choice.

### BERT feature extraction

As illustrated in Figure 1, we adopt the BERT model for peptide sequences feature encoding. To begin with, we consider each molecular sequence in the benchmark dataset as a sentence in the text field with each amino acid being treated as a word within that sentence. And then these peptide sequences are fed into a pre-trained BERT model that consists of four encoder layers, each with eight attention heads and 512 hidden layers. More specifically, it involves two operations: sequence embedding and feature representations acquiring by the encoder layers. Among them, the first step necessitates the addition of [CLS] and [SEP] tokens to each peptide sequence for the purpose of proper embedding of input sequences into the BERT model. While BERT generates corresponding token embedding, segment embedding and position embedding for each amino acid in this process, enabling the model to comprehend contextual information(i.e. the relationships among different amino acids) within the sequence. The task undertaken by another step involves utilizing encoder layers to process those embedding vectors. It is noteworthy that each encoder layer depends on its internal multi-head self-attention and feed-forward neural network sub-layers to effectively convert the embedding vectors of the input sequences into richer feature representations. Regarding the multi-head self-attention sub-layer, it is utilized to perform attention calculation at each position within each input sequence so as to capture the relationships between that position and all other positions. This enables the model to simultaneously consider various parts of the sequence, thereby acquiring contextual information. The specific process of attention calculation [36] is outlined by Formula (1): 


(1) \begin{align*}\left\{\begin{array}{@{}c} \begin{aligned} & \operatorname{MultiHead}(Q, K, V) =\operatorname{Concat}\left(\operatorname{head}_{1}, \ldots, \operatorname{head}_{\mathrm{h}}\right) W^{O} \\[3pt] & \operatorname{head}_{i}=\operatorname{Attention}\left(Q W_{i}^{Q}, K W_{i}^{K}, V W_{i}^{V}\right) \\[3pt] & \operatorname{Attention}(Q, K, V)=\operatorname{softmax}\left(\frac{Q K^{T}}{\sqrt{d_{k}}}\right) V \end{aligned}\end{array}\right.\end{align*}

where Q, K and V, respectively, represent the Q (uery), K(ey) and V(alue) matrices that are calculated from distinct linear transformations of input sequences. $W_{i}^{Q}$, $W_{i}^{K}$ and $W_{i}^{V}$ mean the weight matrices. $d_{k}$ denotes the dimension of the key vector for each attention head in the K(ey) matrix.

For the feed-forward neural network sub-layer, it performs nonlinear transformations on the contextual representation of each position. This sub-layer typically comprises an activation function (such as ReLU) and two linear transformations. Its computational process [36] is presented as Formula (2): 


(2) \begin{align*}& \operatorname{Output}(x)=\max \left(0, x W_{1}+b_{1}\right) W_{2}+b_{2}\end{align*}

where $W_{1}$ and $W_{2}$, respectively, indicate the weight parameters, while $b_{1}$ and $b_{2}$ represent the bias terms and x denotes the representation obtained after undergoing processing through the multi-head self-attention sub-layer.

In addition to these two sub-layers, there is also an important component LayerNorm(x+Sublayer(x)) in the encoder layer. It is used to implement residual connection and layer normalization, aiding in stabilizing model training and learning sequence representations more effectively. Eventually, the final feature representations from the last encoder layer output of the BERT model serve as the input for the subsequent CNN-BiLSTM-Attention approach mentioned.

### CNN-BiLSTM-Attention model construction

In this study, we construct a framework comprised of CNN, BiLSTM and attention mechanism to augment its comprehension and classification power pertaining to AMPs sequences. CNN has the capability to identify patterns within segments of sequences of varying lengths. It also can extract local features from the original features and construct more advanced representations. Taking advantage of the characteristics of CNN, we employ a one-dimensional convolutional layer to perform convolution operations on the features extracted by BERT with the aim of further capturing local patterns and features within the sequences. The details of this convolutional layer are as follows: it is consisted of 64 filters with kernel size one and the ReLU activation function is utilized. Subsequently, the output of CNN is fed into a BiLSTM layer.

BiLSTM is capable of capturing temporal dependencies within sequences while simultaneously considering both forward and backward information so as to generate more comprehensive feature representations. Thereby, BiLSTM models the long-term dependencies and global contextual information of the peptide sequences based on the output of CNN. In this work, we employ a BiLSTM layer which is consisted of two LSTM layers with 64 neurons each in both the forward and backward directions.

Afterwards, the attention mechanism is applied to weight the output of BiLSTM layer by calculating attention weights. The computational process of attention weights is shown in Formula (3): 


(3) \begin{align*}& A_{t}=\operatorname{softmax}\left(\operatorname{Dense}\left(X_{t}\right)\right)\end{align*}

where $X_{t}$ is the input data slicing at time step t.

This mechanism enables the model to focus more on information fragments that play a crucial role in specific tasks when processing input data but disregarding less significant parts. The ability of CNN-BiLSTM-Attention model to effectively capture pivotal features and information within sequences data can be enhanced by introducing this custom-designed attention mechanism in this study, consequently helping the iAMP-Attenpred predictor in achieving elevated accuracy in the AMPs classification tasks.

### Classification module

As depicted in Figure 1, a classification module which includes a flatten layer followed by multiple fully connected layers is used to solve the ultimate binary prediction problem for AMPs. Concretely speaking, the flatten layer transforms the multi-dimensional features obtained from the output of CNN-BiLSTM-Attention model into a one-dimensional vector for the purpose of furnishing a flattened input for the subsequent fully connected layers. Then four fully connected layers with neuron counts of 256, 128, 64 and 2, respectively are sequentially employed to learn more advanced feature representations. It should be noted that the ReLU activation function and a dropout layer with a dropout rate of 0.1 are applied after each fully connected layer except for the final one. Eventually, the sigmoid activation function is employed to output corresponding class probabilities.

### Performance evaluation metrics

To assess the classification performance of iAMP-Attenpred predictor, we choose to calculate seven metrics: sensitivity (SEN), specificity (SPE), the Matthew’s correlation coefficient (MCC), accuracy (ACC), Precision (PRE), F_score (FSC) and the area under ROC (AUROC). Seven indicators are defined as Formula (4): 


(4) \begin{align*}& \left\{\begin{array}{c} \mathrm{SEN}=\displaystyle\frac{\mathrm{TP}}{\mathrm{TP}+\mathrm{FN}} \\[6pt] \mathrm{SPE}=\displaystyle\frac{\mathrm{TN}}{\mathrm{TN}+\mathrm{FP}} \\[6pt] \mathrm{MCC}=\displaystyle\frac{(\mathrm{TP} \times \mathrm{TN})-(\mathrm{FP} \times \mathrm{FN})}{\sqrt{(\mathrm{TP}+\mathrm{FP})(\mathrm{TP}+\mathrm{FN})(\mathrm{TN}+\mathrm{FP})(\mathrm{TN}+\mathrm{FN})}} \\[6pt] \mathrm{ACC}=\displaystyle\frac{\mathrm{TP}+\mathrm{TN}}{\mathrm{TP}+\mathrm{TN}+\mathrm{FP}+\mathrm{FN}} \\[6pt] \mathrm{PRE}=\displaystyle\frac{\mathrm{TP}}{\mathrm{TP}+\mathrm{FP}} \\[6pt] \mathrm{FSC}=\displaystyle\frac{2 \times \mathrm{SEN} \times \mathrm{PRE}}{\mathrm{SEN}+\mathrm{PRE}} \\[6pt] \mathrm{AUROC}: \text{Area under the ROC Curve} \end{array}\right.\end{align*}

where TP and TN, respectively, indicate the number of AMPs and non-AMPs correctly identified by the predictor. FP and FN specifically denote the number of AMPs and non-AMPs that cannot be correctly identified by the predictor. ROC means the receiver operating characteristic curve.

## RESULTS AND DISCUSSION

The 10-fold cross validation approach is employed for reliably estimating the performance of our iAMP-Attenpred predictor in this work. Specifically speaking, the benchmark dataset is split into ten equally sized subsets with nine subsets used for model training and the remaining one subset served as the testing data for performance evaluation of the model. This process is repeated 10 times with different test subsets chosen for each repetition.

### Performance comparison of iAMP-Attenpred predictor and various deep learning methods

In this section, the same benchmark dataset1 presented in Table 1 is applied and the identical BERT feature extraction model is utilized for investigation so as to demonstrate the better classification capacity of the iAMP-Attenpred predictor compared to various deep learning methods. These deep learning models used for comparison include CNN, LSTM, BiLSTM, CNN-LSTM, CNN-BiLSTM and CNN-LSTM-Attention. The final performance comparison results are listed in Table 2. It is evident that the proposed iAMP-Attenpred classification model exhibits more significant performance over the aforementioned deep learning approaches in terms of the four metrics: ACC, MCC, SEN and FSC. In addition, we notice that the PRE and FSC indicators of iAMP-Attenpred method are not significantly distant from those of the classifiers in Table 2 with the highest PRE and FSC values.

**Table 2:** Performance comparison results of iAMP-Attenpred predictor and various deep learning approaches

Predictor	ACC	MCC	SEN	SPE	PRE	FSC
BERT-CNN	0.9758	0.9516	0.9699	0.9811	0.9786	0.9741
BERT-LSTM	0.9781	0.9564	0.9668	0.9881	0.9864	0.9763
BERT-BiLSTM	0.9789	0.9577	0.9730	0.9841	0.9819	0.9773
BERT-CNN-LSTM	0.9774	0.9548	0.9716	0.9826	0.9803	0.9758
BERT-CNN-BiLSTM	0.9765	0.9531	0.9670	0.9849	0.9826	0.9745
BERT-CNN-LSTM-Attention	0.9842	0.9684	0.9768	0.9907	0.9891	0.9828
BERT-CNN-BiLSTM-Attention^*a*^	0.9844	0.9688	0.9813	0.9872	0.9853	0.9833

$^{a}$ It denotes our iAMP-Attenpred predictor.

We conduct an analysis of the performance enhancement, which can be attributed to the following three factors. The first and second factors are that the iAMP-Attenpred predictor incorporates the characteristics of both CNN and BiLSTM, enabling it to effectively capture both local features and bidirectional dependencies in peptide sequence data. The third factor is associated with the attention mechanism that can focus on critical information parts. Therefore, the combination of these three factors leads to the improvement in the predictive performance of the iAMP-Attenpred model.

### Performance comparison of attention mechanism and several machine learning technologies

**Figure 4:**
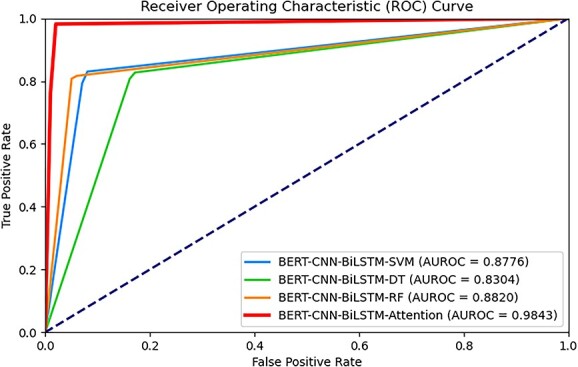
ROC curves generated by iAMP-Attenpred predictor and its variants based on existing machine learning algorithms.

In order to illustrate the importance of the attention mechanism on the iAMP-Attenpred predictor, three variants are constructed based on this predictor to compare performance with it on the benchmark dataset1. All three variants are obtained through modifications using machine learning techniques, wherein the attention mechanism within our classifier is respectively substituted with SVM, DT and RF algorithms. Table 3 presents the ACC, MCC, SEN, SPE, PRE and FSC metrics of the iAMP-Attenpred identification model and its three variants. Moreover, their ROC curves and AUROC indicators are shown in Figure 4. It is not difficult to discover that our new design attains the highest values across all metrics in comparison to the other three variants. As a result of our experiments we conclude that the attention mechanism is a useful mean for the classification ability enhancement of iAMP-Attenpred predictor.

**Table 3:** Performance comparison results of iAMP-Attenpred predictor and its variants based on existing machine learning algorithms

Predictor	ACC	MCC	SEN	SPE	PRE	FSC
BERT-CNN-BiLSTM-SVM	0.8798	0.7618	0.8294	0.9261	0.9118	0.8681
BERT-CNN-BiLSTM-DT	0.8306	0.6609	0.8264	0.8346	0.8207	0.8234
BERT-CNN-BiLSTM-RF	0.8850	0.7745	0.8150	0.9490	0.9364	0.8712
BERT-CNN-BiLSTM-Attention^*a*^	0.9844	0.9688	0.9813	0.9872	0.9853	0.9833

$^{a}$ It denotes our iAMP-Attenpred predictor.

We delineate the explanation for the capacity improvement into two aspects. On the one hand, these traditional machine learning algorithms are typically better suited for structurally simple data so that they might struggle to effectively handle the intricate correlations within the sequences data. In the AMPs classification tasks, the order and interactions within sequences are crucial for accurate prediction, which conventional methods often find challenging to capture. On the other hand, the attention mechanism enables the model to assign varying weights to different parts of the input sequences. In the context of AMPs classification, certain specific amino acid sequence segments might hold greater significance for antimicrobial efficacy. The attention mechanism allows the model to focus more on these pivotal segments, thereby enhancing its ability to capture crucial information.

### Performance assessment of iAMP-Attenpred predictor based on multiple repetitions of 10-fold cross validation

In this study, multiple repetitions of 10-fold cross validation and different datasets are utilized for the purpose of more accurately and reliably assessing the performance and generalization ability of our new predictor. Concretely speaking, we select the benchmark dataset1 and the benchmark dataset2 as illustrated in Table 1 and subsequently perform a comprehensive evaluation of our novel predictive model by conducting multiple repetitions of 10-fold cross validation based on these datasets. The reason we verify it in this way is because this cross validation process can be repeated many times on different subset combinations to reduce accidental errors and ensure a more objective assessment of the evaluation results. Instead, we only present the results of five repetitions of 10-fold cross validation in the table due to space constraints in the article. Tables 4 and 5, respectively, display the performance results of our classifier from five instances of 10-fold cross validation on these two datasets, along with their average performance. It is clearly obvious that the performance metrics from each iteration of 10-fold cross validation are similar to their respective average performance indicators whether for the benchmark dataset1 or the benchmark dataset2. The results of the experiment reveal the consistency and generalization capability of our identification model, indicating its reliability for AMPs classification.

**Table 4:** Five times and average performance results of 10-fold cross validation method based on the benchmark dataset1

NoT^*a*^	ACC	MCC	SEN	SPE	PRE	FSC
1	0.9844	0.9688	0.9813	0.9872	0.9853	0.9833
2	0.9836	0.9673	0.9788	0.9879	0.9861	0.9823
3	0.9838	0.9676	0.9770	0.9897	0.9880	0.9823
4	0.9834	0.9668	0.9777	0.9884	0.9867	0.9820
5	0.9840	0.9681	0.9805	0.9872	0.9855	0.9829
Average	0.9838	0.9677	0.9791	0.9881	0.9863	0.9826

$^{a}$ NoT means number of times.

**Table 5:** Five times and average performance results of 10-fold cross validation method based on the benchmark dataset2

NoT^*a*^	ACC	MCC	SEN	SPE	PRE	FSC
1	0.9766	0.9391	0.9392	0.9902	0.9707	0.9532
2	0.9763	0.9411	0.9579	0.9831	0.9569	0.9562
3	0.9775	0.9430	0.9609	0.9835	0.9558	0.9580
4	0.9802	0.9496	0.9652	0.9856	0.9609	0.9625
5	0.9775	0.9436	0.9634	0.9827	0.9547	0.9586
Average	0.9776	0.9433	0.9573	0.9850	0.9598	0.9577

$^{a}$ NoT means number of times.

### Performance comparison of iAMP-Attenpred predictor and the state-of-the-art classifiers

For the sake of further demonstrating the effectiveness of our new design, we compare it with the other state-of-the-art classifiers. Notably, these existing predictors that also apply computational methodologies to identify AMPs based on the benchmark dataset1 and the benchmark dataset2 are, respectively, iAMP-2L [23], MLAMP [48] and iAMP-CA2L [43]. Among them, the first predictor is built on the PseAAC feature representation method and the fuzzy KNN technology for the purpose of recognizing AMPs. The second predictor and the third predictor are individually developed with the RF approach and the combination model which is relevant with CNN, BiLSTM and SVM methods. Additionally, we utilize average performance metrics of the iAMP-Attenpred predictor as listed in Tables 4 and 5 for a fairer performance comparison. The experimental comparison results between iAMP-Attenpred predictor and the other classifiers based on the different datasets are presented in Tables 6 and 7, from which we can clearly notice that our new design remarkably outperforms the other state-of-the-art classifiers in terms of ACC, MCC, SEN, SPE, PRE and FSC. Especially, our method outperforms other classifiers by approximately 4.25–13% for the benchmark dataset1 and about 4.68–11% for the benchmark dataset2 in terms of ACC metric. This further illustrates our new design can serve as an effectual mean for aiding AMPs discovery.

**Table 6:** Performance comparison results of iAMP-Attenpred predictor and existing methods based on the benchmark dataset1

Predictor	ACC	MCC	SEN	SPE	PRE	FSC
iAMP-2L^*a*^	0.8547	0.7095	0.8897	0.8167	0.8408	0.8646
MLAMP^*a*^	0.8929	0.7863	0.9086	0.8778	0.8766	0.8923
iAMP-CA2L^*a*^	0.9413	0.8829	0.9547	0.9277	0.9310	0.9427
iAMP-Attenpred	0.9838	0.9677	0.9791	0.9881	0.9863	0.9826

$^{a}$ These data were obtained from the original articles.

**Table 7:** Performance comparison results of iAMP-Attenpred predictor and existing methods based on the benchmark dataset2

Predictor	ACC	MCC	SEN	SPE	PRE	FSC
iAMP-2L^*a*^	0.8632	0.7265	0.8713	0.8603	–^*b*^	–
MLAMP^*a*^	0.8990	0.7370	0.7700	0.9460	–^*b*^	–
iAMP-CA2L^*a*^	0.9308	0.8620	0.9458	0.9161	0.9163	0.9308
iAMP-Attenpred	0.9776	0.9433	0.9573	0.9850	0.9598	0.9577

$^{a}$ These data were obtained from the original articles.

$^{b}$ ’-’ means that there is no value in the corresponding item.

## LIMITATIONS

Although iAMP-Attenpred predictor demonstrates superior performance in predicting AMPs, it is important to acknowledge the presence of several limitations in this study. Firstly, our work does not consider more specific information such as the presence of di sulfide bonds, the secondary or tertiary structure of AMPs or posttranslational modifications, which can also be crucial factors influencing AMPs function and activity. We will consider including di sulfide bonds information as an additional input, explore the possibility of including information about posttranslational modifications as an additional input and consider incorporating more structural information into analysis in future study. Secondly, our work currently only has a verifiable computing model and lacks a user-friendly web server. In future work, we plan to develop an intuitive and user-friendly server to enhance the practicality of our method, making it accessible to a broader user group.

## CONCLUSION

In order to effectively enhance the predictable ability of AMPs, a novel predictor named iAMP-Attenpred is proposed in this work. To the best of our knowledge, this new design is the first work that not only leverages BERT feature encoding technique from the NLP field but also integrates various deep learning methods to construct a composite model for AMPs identification. Following the preprocessing of the benchmark datasets, BERT model is employed for feature extraction with the aim of better capturing the structural characteristics of peptide sequences in this study. In addition, a composite model that consists of CNN, BiLSTM and attention mechanism is constructed to learn the distinctive features obtained from BERT approach. Eventually, the classification module composed of a flatten layer and different fully connected layers is adopted to discriminate whether a peptide sequence belongs to the category of AMPs or non-AMPs based on the output of the CNN-BiLSTM-attention model. The results of experiment illustrate that our identification model remarkably outperforms the other existing classifiers on the two different benchmark datasets in terms of ACC, MCC, SEN, SPE, PRE and FSC. Consequently, we can draw a conclusion that the iAMP-Attenpred predictor can aid researchers in achieving more precise and accurate recognition of AMPs. We have reason to believe that the proposed identification model based on the BERT architecture, neural network model and attention mechanism holds potential for extensive applicability across the other various biological sequence analysis challenges.

**Key Points**

- The iAMP-Attenpred predictor based on a combination of multiple deep learning methods including BERT, CNN, BiLSTM and attention mechanism is proposed for the first time to predict and classify antimicrobial peptides sequences.
- Each amino acid from the preprocessed benchmark dataset sequences is treated as a word and then inputted into BERT pre-training model for feature encoding.
- A composite model composed of one-dimensional CNN, BiLSTM and attention mechanism is utilized for learning more discriminate features and a classification module consisted of a flatten layer and various fully connected layers is used to for the final discovery of AMPs.
- Compared to the state-of-the-art antimicrobial peptides prediction methods, our new design achieves the highest sensitivity, specificity, the Matthew’s correlation coefficient, accuracy, precision and F_score. The iAMP-Attenpred prediction model can provide valuable reference and help for researchers in the field of bioinformatics to study antimicrobial peptides.

## Data Availability

The source code and data of iAMP-Attenpred model are publicly available at https://github.com/xingwxzz /iAMP-Attenpred. The files of this program include the benchmark datasets, the source code of data preprocessing, the source code of BERT feature encoding, the source code of training model and so on.

## Author Biographies

**Wenxuan Xing** is a master student majoring in computer technology at the School of Computer Science and Engineering, Northeastern University, Shenyang, China. Her research direction is bioinformatics.

**Jie Zhang** is an associate professor at the College of Computer and Information Engineering, Inner Mongolia Agricultural University, Hohhot, China.

**Chen Li** is a master student majoring in artificial intelligence at the School of Computer Science and Engineering, Northeastern University, Shenyang, China.

**Yujia Huo** is an undergraduate student at the College of Computer and Information Engineering, Inner Mongolia Agricultural University, Hohhot, China.

**Gaifang Dong** is a professor at the College of Computer and Information Engineering, Inner Mongolia Agricultural University, Hohhot, China. Her research direction is bioinformatics.
